# Microstructural White Matter Tissue Characteristics Are Modulated by Homocysteine: A Diffusion Tensor Imaging Study

**DOI:** 10.1371/journal.pone.0116330

**Published:** 2015-02-18

**Authors:** Jung-Lung Hsu, Wei-Hung Chen, Chyi-Huey Bai, Jyu-Gang Leu, Chien-Yeh Hsu, Max A. Viergever, Alexander Leemans

**Affiliations:** 1 Graduate Institute of Biomedical Informatics, College of Medical Science and Technology, Taipei Medical University, Taipei, Taiwan; 2 Section of Dementia and Cognitive Impairment, Department of Neurology, Chang Gung Memorial Hospital, Linkou, Taiwan; 3 Image Sciences Institute, University Medical Center Utrecht, Utrecht, The Netherlands; 4 Department of Neurology, Shin Kong Wu Ho-Su Memorial Hospital, Taipei, Taiwan; 5 Department of Public Health, College of Medicine, Taipei Medical University, Taipei, Taiwan; 6 College of Medicine, Fu Jen Catholic University, Taipei, Taiwan; Beijing Normal University, CHINA

## Abstract

Homocysteine level can lead to adverse effects on the brain white matter through endothelial dysfunction, microstructural inflammation, and neurotoxin effects. Despite previously observed associations between elevated homocysteine and *macroscopic* structural brain changes, it is still unknown whether *microstructural* associations of homocysteine on brain tissue properties can be observed in healthy subjects with routine MRI. To this end, we investigated potential relationships between homocysteine levels and microstructural measures computed with diffusion tensor imaging (DTI) in a cohort of 338 healthy participants. Significant positive correlations were observed between homocysteine levels and diffusivity measures in the bilateral temporal WM, the brainstem, and the bilateral cerebellar peduncle. This is the first study demonstrating that DTI is sufficiently sensitive to relate microstructural WM properties to homocysteine levels in healthy subjects.

## Introduction

The blood level of homocysteine, a sulfur-containing amino acid derived from dietary methionine, is known to increase with age, hypertension, smoking, and renal failure [1–3]. Serum total homocysteine level is associated with vascular risk factors such as hypertension, current smoking, previous cardiovascular disease, which are the composition items of the Framingham stroke risk profile [4, 5]. Homocysteine level may reflect the endothelial dysfunction and is related to inflammation response [4, 6, 7]. While severe hyperhomocysteinemia is rare, mild elevation of blood homocysteine level has been reported to occur in 5–7% of the general population [8]. Previous research suggests that there is a relationship between elevated homocysteine levels and brain morphological factors derived from magnetic resonance imaging (MRI), such as brain atrophy [9], reduced white matter (WM) volume [10], and WM hyperintensity (WMH) abnormalities [11, 12]. Only a few studies did not find any relationship between homocysteine levels and MRI markers [13, 14].

While associations between elevated homocysteine levels and *macroscopic* structural brain changes have been found, it is still unknown whether the *microstructural* brain tissue properties are affected in healthy subjects. Since such microstructural relationships may provide early and sensitive indicators of tissue abnormalities before the manifestation of gross morphological changes, we performed a baseline, cross-sectional study to explore the association between homocysteine level and WM microstructural organization in a large cohort of 338 healthy adults. To this end, diffusion tensor imaging (DTI) [15–17] data were acquired from an ethnically homogenous Asian population, in which subjects were carefully screened for medical and neurological conditions minimizing sources of variation that could affect the sensitivity of our imaging findings. In particular, we investigated whether the level of homocysteine was associated with specific diffusivity properties while controlling for various vascular risk factors.

## Materials and Methods

### Subjects

The subjects in this study were recruited from a health-screening program in Taiwan. In total, 357 participants, aged 25–81 years, received detailed examinations, including physical and bed-side neurological examinations performed by neurologists, a biochemistry study, a chest X-ray, an electrocardiogram (EKG), and MRI scans. The blood tests included the assessment of hemoglobulin (Hb), mean cell volume, fasting glucose, creatinine, high-sensitivity CRP (hs-CRP), and total level of homocysteine. All participants were asked to fast overnight (≥ 8 hours) before blood samples were taken. The blood samples were analyzed using colorimetry by an automatic chemistry analyzer in an approved laboratory (UniCel DxC 800; Beckman Coulter). The level of homocysteine was determined with a standardized chemiluminescent microparticle immunoassay (CMIA) using the ARCHITECT *i* system (Abbott Laboratories, Abbott Park, IL). For each individual the body mass index (BMI) and Framingham Stroke Risk Profile (FSRP) score were also computed [5, 18]. Participants who had a history of major neurological diseases, cerebrovascular accidents, or those with manifestations of stroke, psychiatric conditions, or serious cardiovascular diseases were excluded. In the end, 338 participants were included for further analysis; 19 subjects were excluded: eleven participants had an ARWMC (age-related white matter changes) score > 2 as derived from the T2-weighted and FLAIR images [19], for seven participants the FSRP was not available, and one participant had an abnormally high homocysteine level of 23.5 μmol/L.

### MRI scans

All participants received whole-brain MRI scans (Siemens, 1.5 T) at the same day of the clinical examinations. First, trans-axial T2-weighted scans, FLAIR images and high-resolution sagittal T1-weighted images were acquired. Next, a whole-brain DTI scans (TR/TE = 7600/82 ms, 3 mm slice thickness without gap, slice acquisition matrix = 128 x 128 with FOV = 256 x 256 mm^2^, 6/8 partial Fourier, NEX = 1, 55 slices, 12 gradient directions with b-value = 1000 s/mm^2^, and one b = 0 s/mm^2^ image) were acquired axially with a fat suppression sequence [16]. The Ethics Committee and Institutional Review Board of Shin Kong Wu Ho-Su Memorial Hospital approved the study. The written informed consent was provided by the participants. Detailed overview of the scanning parameters can be found in previous literature [20].

### DTI processing

The DTI based processing steps performed in this work have been described previously in detail [20, 21]. In summary, the following steps were taken:

- All DTI data sets were corrected for eddy current induced geometric distortions and subject motion [22].
- The diffusion tensor model was fitted to the data with *ExploreDTI* (http://www.exploredti.com)[23] using a non-linear regression method. Fractional anisotropy (FA) and the mean (MD), axial (AD) and transverse (TD) diffusion measures were subsequently computed [24]. Note that these DTI based measures have been shown to be more sensitive to tissue abnormalities than the typical visual evaluation of WM hyperintensities observed in conventional MRI data [25–27].
- A population-based DTI atlas in MNI space was constructed [20, 28] to drive the tensor based affine [29] and non-affine [30] coregistration techniques. At the final transformation step, the “preservation of principal direction” strategy was applied to reorient the diffusion tensor [31].

For each individual, the intracranial volume in native space was extracted from the b = 0 s/mm^2^ image with BET2, the brain extraction tool from FSL (http://www.fmrib.ox.ac.uk/fsl/) [32]. The intracranial volume was further subdivided into its CSF regions and brain tissue (combined global GM and WM) by segmenting all CSF voxels using an automated gray-level thresholding method, performed on the MD map [33]. For each subject, the mean FA, MD, AD, and TD values within brain tissue were calculated for further analysis.

### Statistical analysis

All data analyses described below were conducted on the 338 participants and reviewed by the medical statistician of our institution. Distributions of FSRP, CRP and ARWMC-total score were positively skewed (p < 0.0001) and thus were subjected to log_10_-transformation to improve normality before being used for statistical model inferences. Pairwise correlation was used to explore the relations between the various clinical measures.

For the global DTI analysis, a linear regression model was used to explore the effect of homocysteine on the mean FA, MD, AD and TD values within the brain (combine global GM and WM volume), controlling for eight covariates (i.e., age, gender, BMI, log(FSRP), log(hs-CRP), Hb, creatinine and log(ARWMC-total score)). Statistical significance was set to p-value < 0.01.

To study the effect of homocysteine in more detail in terms of brain localization (i.e., which brain regions are involved), we also performed a voxel based analysis (VBA)[34] with spatially normalized DTI datasets (using the FA, MD, AD and TD maps, voxel size: 2x2x2 mm^3^, and FWHM = 6 mm Gaussian smoothing) [34]. To explore the effect of homocysteine on FA, MD, AD and TD measures, multiple regression models with the abovementioned eight covariates were used. The resulting T-statistic images were subsequently thresholded for significance: regions with family-wise error (FWE) corrected p-value < 0.01 with corresponding T-values above 5.2 and with cluster sizes larger than 200 voxels (voxel size: 2x2x2 mm^3^) were considered significant after correction for multiple independent comparisons. The significant regions were superimposed on the standardized MNI T1-weighted image and on the main fiber bundles, reconstructed from DTI template developed by Mori et al [35].

## Results

### Descriptive statistics for clinical measures

Clinical and demographic characteristics of the 338 participants are shown in Table 1. In summary, the mean age of the study participants is 51.6 years and 41.4% of them were female. The mean FSRP score is 4.9%. The mean value of homocysteine is 8.6 μmol/L (range: 3.4–16.2 μmol/L) and only four participants (1.2%) have a homocysteine level above 15 μmol /L. Males have a higher homocysteine level than females (males: 9.6 ± 2.3 μmol /L v.s. females: 7.3 ± 1.9 μmol /L, p < 0.0001). The median ARWMC-total score is 0 (range: 0–8) and only twenty participants (5.8%) have an ARWMC-total score larger than 2, which reflects the beginning confluence of WM lesions at that particular brain region.

**Table 1:** Characteristic of the study participants (N = 338).

Variable	value
Age in years, mean (SD)	51.6 (10.5)
Age ≥ 65 years, n (%)	37 (10.9)
Female, n (%)	140 (41.4)
Current smoker, n (%)	69 (20.3)
Hypertension, n (%)	80 (23.6)
Diabetes mellitus, n (%)	36 (10.7)
History of cardiovascular disease, n (%)	16 (4.7)
EKG-atrial fibrillation, n (%)	1 (0.3)
EKG-LVH, n (%)	27 (7.9)
FSRP score (%), mean (SD)	4.9 (5.1)
Systolic BP (mm Hg), mean (SD)	121.5 (20.3)
Diastolic BP (mm Hg), mean (SD)	73.3 (11.8)
BMI (kg/m^2^), mean (SD)	24.2 (3.2)
Hb (gm/dl), mean (SD)	14.5 (1.5)
Fasting blood glucose (mg/dl), mean (SD)	93.9 (18.1)
hs-CRP (mg/dl), mean (SD)	0.3 (0.5)
Total homocysteine (μmol /L), mean (SD)	8.6 (2.4)
Creatinine (mg/dl), mean (SD)	0.8 (0.2)
ARWMC-total score, median	0
MRI volumetric measures	
FA, mean (SD)	0.26 (0.01)
MD (x10^–5^ mm^2^/s), mean (SD)	76.8 (3.2)
AD (x10^–5^ mm^2^/s), mean (SD)	97.7 (4.0)
TD (x10^–5^ mm^2^/s), mean (SD)	66.5 (2.9)

EKG: electrocardiogram; LVH: left ventricular hypertrophy; FSRP: Framingham Stroke Risk Profile; BP: blood pressure; BMI: body mass index; Hb: hemoglobin; hs-CRP: high-sensitive C-reactive protein; ARWMC: Age-related WM changes; FA: fractional anisotropy; MD: mean diffusivity; AD: axial diffusivity; TD: transverse diffusivity.

Pairwise correlations between age, gender, BMI, FSRP score, total level of homocysteine, hs-CRP, Hb, creatinine and ARWMC-total score are shown in Table 2. In summary, the level of homocysteine is positively correlated with BMI, FSRP score, Hb and creatinine.

**Table 2:** Pairwise correlation matrix of clinical measures in the participants.

	Gender	BMI	Log(FSRP score)	Total homocysteine	Log(hs-CRP)	Hb	Creatinine	Log(ARWMC-total score)	FA	MD	AD	TD
Age	-0.04	0.10	0.41*	0.14	0.15*	-0.13	0.03	0.37*	-0.24*	0.32*	0.25*	0.36*
Gender		0.22*	0.60*	0.45*	0.11	0.68*	0.56*	-0.06	0.16*	0.10	0.12	0.08
BMI			0.27*	0.14*	0.35*	0.16*	0.11	0.07	0.09	0.01	0.01	0.01
Log(FSRP score)				0.37*	0.16*	0.37*	0.37*	0.26*	-0.09	0.34*	0.30*	0.35*
Total homocysteine					0.11	0.28*	0.34*	0.01	0.08	0.33*	0.36*	0.31*
Log(hs-CRP)						0.06	-0.03	0.06	0.08	0.02	0.02	0.01
Hb							0.36*	-0.09	0.16*	-0.03	-0.01	-0.04
Creatinine								-0.01	0.07	0.03	0.03	0.03
Log(ARWMC-total score)									-0.24*	0.29*	0.24*	0.32*
FA										-0.31*	-0.09	-0.45*
MD											0.97*	0.99*
AD												0.92*

Data values represent the correlation coefficients.

Gender is coded by male: 1 and female: 0.

Log(FSRP score): log-transformed FSRP score; log(hs-CRP): log-transformed hs-CRP; log(ARWMC-total score): log-transformed ARWMC-total score.

For other abbreviations, see Table 1.

* p < 0.01.

### Global analysis

In the global analysis of the DTI images, homocysteine level correlates positively with the mean brain MD, AD and TD (whole model R^2^ = 0.28, 0.24 and 0.29 respectively, all p-values < 0.0001) while adjusted for multiple covariates, including the vascular risk burden (measured by FSRP score) and WM lesions (measured by ARWMC-total score).

### Voxel based analysis

**Fig 1:**
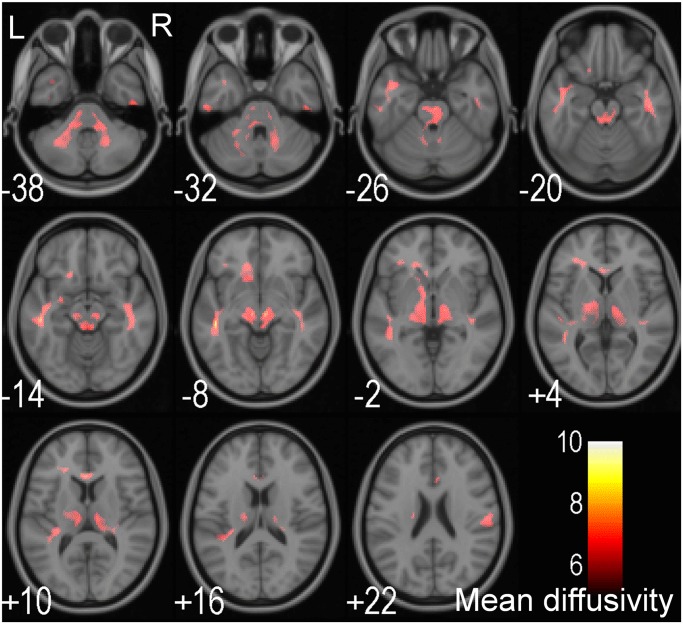
T-value significance maps of the association between homocysteine and mean diffusivity (MD). voxel-based DTI analysis showing a significant positive correlation between the level of homocysteine and MD in the bilateral cerebellar peduncles, the brainstem, the bilateral anterior temporal WM, and the genu of the corpus callosum (in red). The number indicates the z-axis coordinate in MNI space (unit in mm). R: right, L: left.

**Fig 2:**
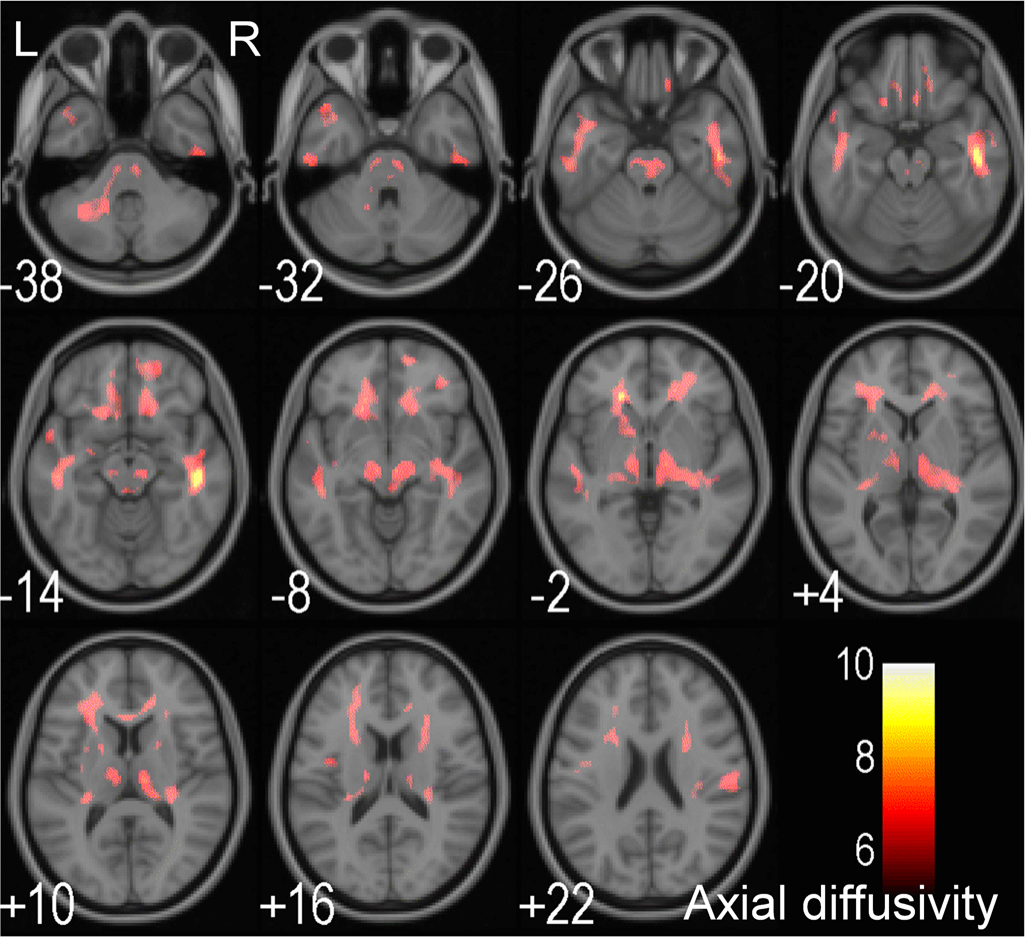
T-value significance maps of the association between homocysteine and axial diffusivity (AD). voxel-based DTI analysis showing a significant positive correlation between the level of homocysteine and the AD in the left cerebellar peduncle, the brainstem, the bilateral anterior temporal WM, and the genu of the corpus callosum (in red). The number indicates the z-axis coordinate in MNI space (unit in mm). R: right, L: left.

In the DTI based VBA approach, there is no significant correlation between the level of homocysteine and FA. Five clusters, which are distributed in the bilateral frontal WM, anterior temporal WM, thalamus, mid brain, middle cerebellar peduncles, the pons, and the genus of the corpus callosum show a significant positive correlation between MD and the level of homocysteine (Fig. 1 and Table 3). In addition, in eight clusters in the bilateral frontal WM, anterior temporal WM, thalamus, mid brain, the left middle cerebellar peduncle, the pons, and the genus of corpus callosum, there is a significant positive correlation between AD and homocysteine (Fig. 2 and Table 4). Finally, homocysteine levels does not show any significant correlation with TD.

**Table 3:** The voxel-based DTI analysis of significant clusters that showed a significant positive correlation with homocysteine and MD.

Structure name	Cluster voxel number	Peak T value	MNI coordinates (mm)		
			X	Y	Z
Temporal WM (L)	1047	7.78	-46	-30	-8
Midbrain (R),	3533	7.30	6	-20	-8
Midbrain (L),		7.26	-8	-20	-6
Temporal WM (R)		7.07	46	-20	-18
Genu of the corpus callosum (L)	592	7.05	-2	26	10
Frontal WM (L)	285	6.93	-20	-4	50
Frontal WM (R)	211	6.63	60	-8	24

MNI: Montreal Neurological Institute; WM: white matter; R: right side; L: left side.

**Table 4:** The voxel-based DTI analysis of significant clusters that showed a significant positive correlation with homocysteine and AD.

Structure name	Cluster voxel number	Peak T value	MNI coordinates (mm)		
			X	Y	Z
Temporal WM (R)	3192	8.32	48	-24	-16
Frontal WM (L)	1769	8.02	-18	30	-4
Temporal WM (L)	1096	7.10	-48	-24	-14
Frontal WM (R)	1378	7.09	18	36	0
Midbrain (L)	650	7.05	-8	-18	-6
Parietal (R)	260	6.96	58	-22	24
Cingulate WM (L)	489	6.93	-14	8	44
Post-central WM (L)	281	6.20	-46	-14	26

MNI: Montreal Neurological Institute; WM: white matter; R: right side; L: left side.

**Fig 3:**
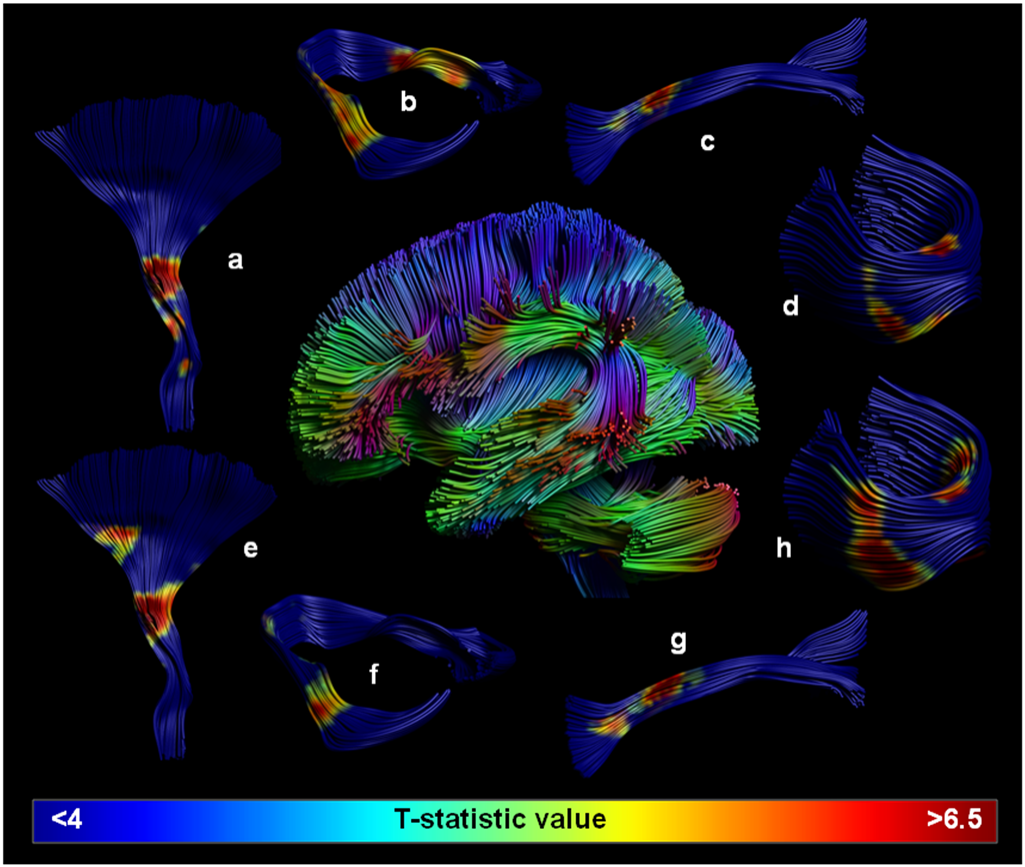
T-value significance maps superimposed on the main white matter tracts. T-value significance maps of the association between homocysteine and mean diffusivity (MD) (a-d) and axial diffusivity (AD) (e-h) were superimposed on the four major white matter tracts that were involved: the pyramidal tracts (a,e), ponto-cerebellar tracts (b,f), inferior longitudinal fasciculus (c,g), and forceps minor (d,h). Color coding from cold to hot represents the T-value derived from the significance maps.

The main regions that showed significant correlations with MD and AD were located in four main WM fiber bundles: the inferior longitudinal fasciculus, the ponto-cerebellar tracts, the forceps minor, and the pyramidal tracts (Fig. 3).

**Fig 4:**
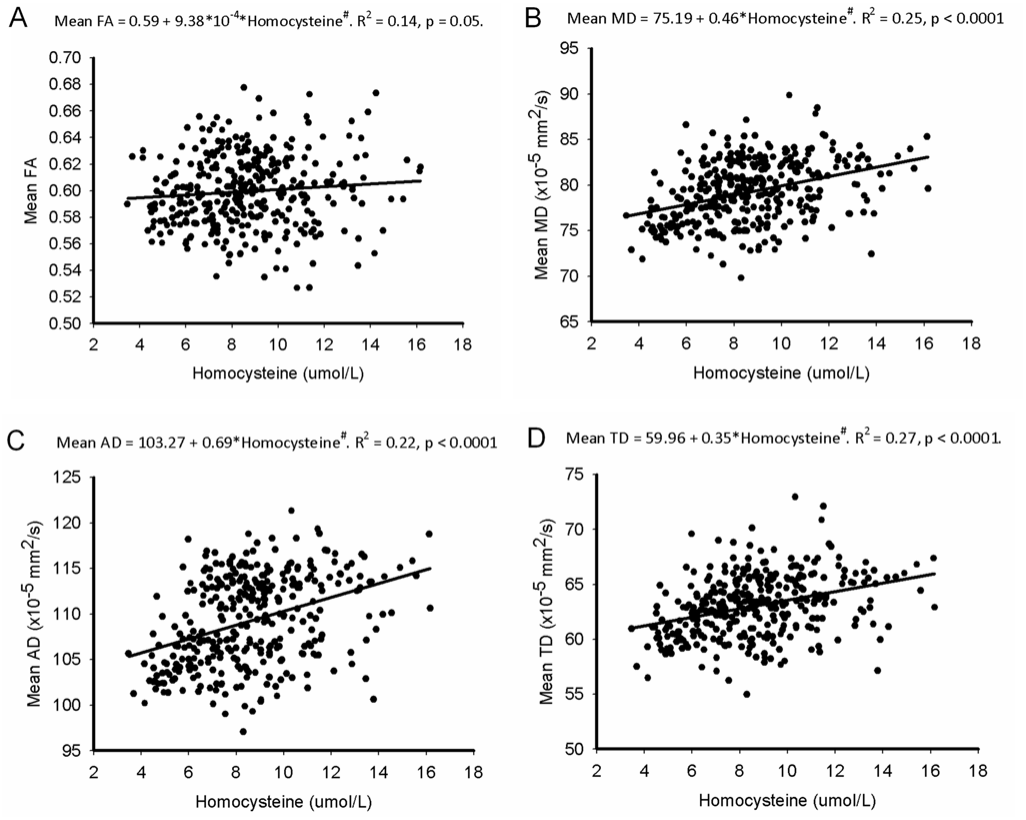
Effect of homocysteine on the fractional anisotropy (FA) (A), mean diffusivity (MD) (B), axial diffusivity (AD) (C), and transverse diffusivity (TD) (D). in contrast with the FA, the MD, AD, and TD are positively correlated with homocysteine. The linear regression model has been adjusted for age, log-transformed FSRP, BMI, log-transformed hs-CRP, hemoglobin, creatinine, log-transformed ARWMC score, and gender. The trend slopes and significance p-values for homocysteine with respect to the DTI measures are indicated.

From the DTI based VBA approach, it is evident that homocysteine shows a widespread positive correlation with MD. To investigate this finding in more detail, we calculated the mean FA, MD, AD and TD values in these regions of significant correlation. We used a linear regression model to explore the effect of homocysteine after adjusting for abovementioned eight covariates. Our results show that homocysteine is positively correlated with mean MD, AD and TD (whole-model p < 0.0001 and homocysteine effect p < 0.0001 for each parameter) (Fig. 4). The beta estimate of homocysteine in the regression model is highest for the AD (AD/MD/TD = 0.69/0.46/0.35, respectively). There is no association between the level of homocysteine and mean FA. The statistical power values for MD, AD and TD in the linear regression model are all above 95%; for the FA it is 53.8%.

## Discussion

In this work, we have investigated the association between homocysteine and DTI parameters in 338 healthy middle-aged participants. Compared to the more widely used morphological and volumetric analyses for studying *macrostructural* brain changes (based on T1-weighted MRI), we used DTI based analyses to probe the *microstructural* tissue properties. In our study design, we carefully controlled for numerous potentially confounding factors such as age, various vascular risk factors (including hypertension, diabetes mellitus, smoking, cardiovascular events history, and EKG features), and cerebral WMH scores.

This is the first study in humans that investigated the association between brain tissue properties derived from DTI and the level of homocysteine. To the best of our knowledge, only one animal study has been performed that has explored this relationship before. In that recent animal study, Willette et al. demonstrated that higher homocysteine levels are associated with lower WM volumes in the pons and the middle cerebellar peduncle, and that higher homocysteine levels have a positive association with the MD in the bilateral anterior cerebellum and the prefrontal regions [36]. They also showed that homocysteine has a significant negative correlation with the FA in the cerebellar WM immediately dorsal to the forth ventricle, which is related to the pontocerebellar fibers. Our findings are in line with their results inasmuch as the MD in the bilateral frontal WM, midbrain, middle cerebellar pedundles, and pons showing a significant positive correlation with the level of homocysteine. More specifically, from VBA analyses, our results demonstrate that the increased MD in the bilateral temporal WM and brain stem regions was mainly caused by an increase in the AD rather than the TD, even when adjusting for multiple vascular risk factors.

While elevated homocysteine levels have been reported in obsessive-compulsive disorder [37, 38], first-episode psychosis [39, 40], schizophrenia [41, 42] and Alzheimer’s dementia [12, 43], no direct link with microstructural properties of the fiber tracts involved has been made previously. In this work, however even within a healthy cohort population, there were significant associations between diffusion metrics and homocysteine levels for the inferior longitudinal fasciculus, the ponto-cerebellar tracts, the forceps minor, and the pyramidal tracts. As these findings represent a baseline reference, it can bring unique insight into the neural substrate of brain disorders.

The biological interpretation of why diffusivity changes are related to homocysteine is far from trivial. In general, diffusion rates are larger if the coherence of tissue organization is compromised. With previous literature indicating that (i) homocysteine can act as an excitatory neurotransmitter leading to oxidative stress, endothelial dysfunction, inflammation, and neuronal injury [44] and (ii) homocysteine is associated with axonal demyelination in periventricular and subcortical WM through the injury of oligodendrocytes [45–47], it is plausible that the observed diffusivity increases were caused by such mechanism of axonal degeneration/injury rather than demyelination [48, 49]. Although hs-CRP and homocysteine are both chronic low-grade inflammation markers [50, 51], only homocysteine showed a significant association in this work, suggesting that homocysteine would have a more specific association with regional cerebral WM microstructural properties than hs-CRP. As such, future studies may benefit from including other inflammation markers such as cytokines to further increase the specificity of regional findings.

Previous work relating macrostructural brain properties with level of homocysteine showed that homocysteine levels are higher with increased WM atrophy or with higher levels of WMH scores [9, 13, 52]. In this work, we do not find a significant association between homocysteine and WMH scores after adjusting for multiple covariates. This observation can be attributed to the fact that the subjects included in this work are relatively young compared to other investigations, and that their average level of homocysteine is relatively low.

There are several methodological considerations in this study. The first one is the cross-sectional nature of our study design. Given that we measured the level of homocysteine only at one time point, we cannot demonstrate any causal effects of homocysteine on brain structure. Secondly, we did not measure the folate or vitamin B_12_ levels of our subjects. Previous work suggests that these measures may modulate the association between homocysteine and structural brain properties and, therefore, should be used as covariates during statistical analysis [10, 11, 52]. However, our participants did not have any history of medical illness, and with a mean hemoglobin equal to 14 mg/dl and the mean volume of red blood cells volume equal to 90 fl, a deficiency of vitamin B_12_ or folate is likely to be absent for the majority of our participants. Furthermore, given that several other studies did not adjust for folate and vitamen B_12_ levels and still found an association between homocysteine level and structural brain properties [9, 36, 47], it is likely that homocysteine can still genuinely affect the WM microstructure [10, 53]. Finally, it should be clear that DTI does not provide direct measures of “WM integrity” (e.g., see recent reviews by [54, 55]. Although DTI is sensitive to microstructural changes, its measures are heavily affected by partial voluming [31, 56] and “crossing fibers” [57–59], which makes it difficult to interpret the results in an unambiguous way.

## Conclusions

We have shown that DTI is sufficiently sensitive to detect associations between homocysteine levels and diffusivity metrics in healthy subjects. Specifically, significant positive correlations were observed between homocysteine levels and diffusivity measures in the bilateral temporal WM, the brainstem, and the bilateral cerebellar peduncle. This is the first study demonstrating that microstructural WM properties are related to homocysteine levels in healthy human subjects.
